# Tackling G × E × M interactions to close on-farm yield-gaps: creating novel pathways for crop improvement by predicting contributions of genetics and management to crop productivity

**DOI:** 10.1007/s00122-021-03812-3

**Published:** 2021-03-18

**Authors:** Mark Cooper, Kai P. Voss-Fels, Carlos D. Messina, Tom Tang, Graeme L. Hammer

**Affiliations:** 1 Queensland Alliance for Agriculture and Food Innovation, The University of Queensland, St Lucia, Brisbane, QLD 4072 Australia; 2 Corteva Agriscience, Research and Development, Johnston, IA 50131 USA

## Abstract

**Key message:**

Climate change and Genotype-by-Environment-by-Management interactions together challenge our strategies for crop improvement. Research to advance prediction methods for breeding and agronomy is opening new opportunities to tackle these challenges and overcome on-farm crop productivity yield-gaps through design of responsive crop improvement strategies.

**Abstract:**

Genotype-by-Environment-by-Management (G × E × M) interactions underpin many aspects of crop productivity. An important question for crop improvement is “How can breeders and agronomists effectively explore the diverse opportunities within the high dimensionality of the complex G × E × M factorial to achieve sustainable improvements in crop productivity?” Whenever G × E × M interactions make important contributions to attainment of crop productivity, we should consider how to design crop improvement strategies that can explore the potential space of G × E × M possibilities, reveal the interesting Genotype–Management (G–M) technology opportunities for the Target Population of Environments (TPE), and enable the practical exploitation of the associated improved levels of crop productivity under on-farm conditions. Climate change adds additional layers of complexity and uncertainty to this challenge, by introducing directional changes in the environmental dimension of the G × E × M factorial. These directional changes have the potential to create further conditional changes in the contributions of the genetic and management dimensions to future crop productivity. Therefore, in the presence of G × E × M interactions and climate change, the challenge for both breeders and agronomists is to co-design new G–M technologies for a non-stationary TPE. Understanding these conditional changes in crop productivity through the relevant sciences for each dimension, Genotype, Environment, and Management, creates opportunities to predict novel G–M technology combinations suitable to achieve sustainable crop productivity and global food security targets for the likely climate change scenarios. Here we consider critical foundations required for any prediction framework that aims to move us from the current unprepared state of describing G × E × M outcomes to a future responsive state equipped to predict the crop productivity consequences of G–M technology combinations for the range of environmental conditions expected for a complex, non-stationary TPE under the influences of climate change.

## Introduction

We use modelling and simulation whenever our empirical experimentation is not feasible because of costs, risks, impacts, or required scale. The complex factorial nature of Genotype-by-Environment-by-Management (G × E × M) interactions in current and future scenarios requires use of modelling and simulation to augment our empirical studies to evaluate many of the important properties of prediction frameworks designed to improve on-farm crop productivity for current and future climates. The proposed integrated empirical-modelling framework provides flexibility to account for contributions from genetics, management and their interactions. The proposed framework requires iterative enhancement of crop models, to account for the potential influences of G × E × M interactions for traits and applications within genomic prediction for breeding (e.g. Technow et al. 2015; Messina et al. 2018; Diepenbrock et al. 2021) and agronomic prediction for optimisation of crop management (e.g. Hammer et al. 2014). Empirical discovery work underpins the definition of targets for improvement of crop models and simulation methods. We discuss how the framework can be applied to design crop improvement strategies to enhance crop productivity and close on-farm yield-gaps in the presence of strong G × E × M interactions. Further, we also consider how the framework and the prediction methods can be applied to account for the non-stationary effects of climate change. Importantly, the iterative empirical-modelling framework is grounded and motivated by successful outcomes achieved from applications to coordinate the development of improved maize hybrids and agronomy for the heterogeneous environments of the US corn-belt (McFadden et al. 2019; Boyer et al. 2013; Cooper et al. 2014a, 2020; Gaffney et al. 2015). The pathway to impact, from proof of concept, through reduction to practice, to adoption for improved maize yield productivity in the water-limited environments of the US corn-belt, was achieved through a long-term research effort involving private–public partnerships (Messina et al. 2020a).

### Seeking workable Genotype–Management technology solutions

Crop scientists are familiar with the concept of Genotype-by-Environment (G × E) interactions and their implications for the on-farm crop productivity achieved by farmers. Plant breeders design breeding programs to account for the effects of G × E interactions in the processes involved in creation and selection of new genotypes with superior performance for a Target Population of Environments (TPE). Agronomists design and evaluate management strategies for the same TPE. These management strategies are designed to work for the genotypes created by the breeders. Farmers adopt the improved genotypes and management strategies to achieve the potential on-farm crop productivity for Genotype–Management (G–M) technology combinations. Farmers must also manage the risk of crop failures (yield and quality) due to the occurrence of extreme environmental conditions. Breeders and agronomists recognise the potential for Genotype-by-Environment-by-Management (G × E × M) interactions (Messina et al. 2009; Snowdon et al. 2020). However, to date the magnitude of the G × E × M factorial has made it impractical to design breeding programs or agronomy research programs to deal with G × E × M interactions explicitly at all stages of the crop improvement process. However, new opportunities are emerging to tackle the complexities of the G × E × M factorial through complementing empirical research programs with an array of prediction methods (Messina et al. 2009, 2020a, b; Cooper et al. 2014a, b, 2020; Chenu et al. 2017; Hammer et al. 2020; Peng et al. 2020; Ersoz et al. 2020; Ramirez-Villegas et al. 2020; Kruseman et al. 2020; Rotili et al. 2020). These prediction methods take advantage of advances in genomics, proximal and remote environmental sensor technologies, phenotyping, envirotyping, and genetic and crop modelling, together with high-performance computing. Therefore, within the scope of crop improvement there is interest in the development of prediction methodologies that can account for important G × E × M interactions that affect current and future crop productivity.

Improving crop productivity and breeding for improved climate resilience is a “big objective” for crop improvement (e.g. Reynolds 2010; Yadav et al. 2011; Chapman et al. 2012; Fischer et al. 2014; Vermeulen et al. 2018; Hammer et al. 2020; Cassman and Grassini 2020; Ramirez-Villegas et al. 2020; Kruseman et al. 2020; Snowdon et al. 2020; Prasanna et al. 2021; Kamenya et al. 2021). Lobell and Burke (2010) posed a fundamental question: “How will climate change interact with the many other factors that affect the future of food production and food security?” As they state, “there are no easy answers”. However, the compelling evidence for climate change (IPCC 2014; National Research Council 2020) leaves no choice but to question the impact of non-stationary changes in the TPE on the requirements for crop adaptation and crop productivity and to seek *workable solutions* that open pathways towards finding answers to their question. Historically, successful *workable solutions*, those that have enabled the documented improvements in on-farm crop productivity (e.g. Smith et al. 2014; Fischer et al. 2014; Snowdon et al. 2020), have been outcomes of positive interactions between genotype (G) and the agronomic management (M) of the selected genotypes, herein referred to as G–M technologies (Hammer et al. 2014), for the range of environments (E) encountered in the TPE. We can anticipate this to be a consistent feature for the foreseeable future. Anthropogenic drivers of climate change impose a directionality to changes in the environmental composition and variability of crop productivity within the TPE, to which we seek workable genotype and management solutions. We can use our collective understanding from the scientific disciplines contributing to crop improvement to predict the interplay of genetics and management with the projected environmental changes associated with climate change to provide a foundation to understand the impact of climate change on the future of food production (Chapman et al. 2012; Messina et al. 2020b; Hammer et al. 2020; Snowdon et al. 2020). At present, breeders and agronomists largely work as a sequential tag team, or independently, conducting empirical research and making predictions to seek answers for the pressing questions within their own domains (e.g. Reynolds 2010; Fischer et al. 2014; Cooper et al. 2014b; Assefa et al. 2018; Edreira et al. 2018; Beres et al. 2020; Rotili et al. 2020; Snowdon et al. 2020). Proposals to accelerate improvement of crop productivity to account for the effects of climate change have largely ignored the influences of G × E × M interactions, or have assumed that the traditional research sequence of breeding for improved adaptation followed by agronomic optimisation will be effective for our future agricultural systems (e.g. Federoff et al. 2010; Reynolds 2010; Yadav et al. 2011; Snowdon et al. 2020). Alternative approaches based on advancing prediction methods to explore and exploit the full potential of the G × E × M state-space are emerging and deserve greater consideration (Chapman et al. 2012; Peng et al. 2020; Messina et al. 2020b). Prediction of G × E × M interactions will require an integration of the progress that has been made to enable genomic prediction of traits for breeding applications, together with advances in understanding crop responses to environmental variation for applications in agronomic prediction (Hammer et al. 2019; Voss-Fels et al. 2019; Messina et al. 2020b). Consideration of the foundations for such prediction of crop productivity outcomes in the presence of G × E × M interactions will be the primary focus of this review.

In theory, whenever G × E × M interactions exist, and they account for a major source of the yield variation among genotypes, there is the potential to select positive G–M technology combinations to accelerate the rate of improvement for on-farm crop productivity (Hammer et al. 2014, 2020). This also creates opportunities to close yield-gaps between the current on-farm yields and the achievable yields (and potential yields), given the environmental conditions (van Ittersum et al. 2013; Fischer et al. 2014; Sadras et al. 2015; Cooper et al. 2020; Rotili et al. 2020). However, in practice, to realise the potential benefits of a crop improvement strategy targeting G–M technology combinations, there must be repeatable positive contributions from the G × M interactions associated with specific G–M technology combinations that can predictably enhance yield productivity in the target on-farm environments (Hammer et al. 2014, 2020; Cooper et al. 2020). Further, beyond prediction, there must be mechanisms for detection and selection of the positive G–M combinations by either or both breeders and agronomists during the crop improvement process (Cooper et al. 2014b; Hammer et al. 2020). Finally, to realise the benefits, farmers must adopt the positive G–M technology combinations for their on-farm crop production systems. Here we consider some motivating examples and discuss these foundations for prediction of crop productivity within the context of G × E × M interactions (Messina et al. 2009, 2020a, b; Kholová et al. 2013, 2014; Ramirez-Villegas et al. 2020; Kruseman et al. 2020).

### Exploiting workable Genotype–Management technology solutions: an example

Long-term improvement of maize production in the US corn-belt provides an illustrative example to understand the hallmarks that enable translation of G × E × M interactions into pathways to improved crop productivity. Elements of the theory and practice for exploiting G–M technology combinations, within the context of G × E × M interactions, as defined above, can be recognised in the well-documented contributions of hybrid-by-density interactions to the historical increase in on-farm yield productivity of maize in the US corn-belt (Duvick et al. 2004, Duvick 2005a, b; Hammer et al. 2009; Assefa et al. 2018). Hybrid-by-density interactions for yield of maize represent an example of repeatable G × M interactions that became targets for selection by breeders and for investigation and optimisation by agronomists. Components of these G × M interactions themselves had predictable interactions with environmental variation for on-farm water availability and nitrogen fertiliser inputs across the US corn-belt environments. Therefore, important G × E × M interactions for yield existed and positive G–M technology combinations, representing repeatable, positive G × M interactions, became workable crop improvement targets. We adopt the notation (G–M) × E to emphasise the definition of components of the total G × E × M interaction that are associated with the interaction of G–M technologies and environmental variation. As genetic variation for tolerance of maize hybrids to high density became better understood, breeders developed methods to successfully select maize hybrids with improved levels of high-density tolerance (Duvick et al. 2004; Duvick 2005a, b; Tollenaar and Wu 1999; Tollenaar and Lee 2002; Lee and Tollenaar 2007). Farmers operating in regions of the US corn-belt with reliable access to water, through either high rainfall or access to irrigation, systematically increased on-farm plant density and nitrogen inputs as hybrids with improved high-density tolerance became available (Duvick et al. 2004). Thus, farmers exploited positive G × M interactions by selecting G–M technology combinations, enabled through appropriate hybrid selection and management of plant density and nitrogen inputs, in combination with their understanding of on-farm environmental water supply conditions. In response to these trends, commercial maize breeders operating in the US corn-belt undertook multi-environment testing of potential new hybrids at higher plant density levels, relative to the distribution of plant density levels used by farmers. Thus, through breeding programs designed to exploit the positive G × M interactions associated with hybrid tolerance to high density, farmers gained access to a range of hybrids that delivered high grain yield for the range of densities they used on-farm, which depended on reliability of access to water and water holding capacity of the soil type. Therefore, the farmers could manage plant density, hybrid selection and other inputs to target on-farm crop yields, conditional on the water availability for their specific environments, and in alignment with their attitudes towards risk of achieving their target level of on-farm crop productivity.

A number of other studies have described the importance of G × E × M interactions and their contributions to crop yield productivity (Cooper et al. 2001; Duvick et al. 2004; Messina et al. 2009; Hatfield and Walthall 2015; Beres et al. 2020; Peake et al. 2020; Rotili et al. 2020; Snowdon et al. 2020). Beyond the hybrid-by-density maize example above, a few studies have demonstrated effective improvement and utilisation of positive G × M interactions: improvement of sorghum yield for drought-prone environments in Australia (Hammer et al. 2014, 2020; Rodriguez et al. 2018); improvement of wheat for drought-prone and irrigated environments in Australia (Hunt et al. 2019); and improvement of maize hybrids for drought-prone environments of the US corn-belt by targeting selection for traits contributing to effective water use (Messina et al. 2009, 2018; Cooper et al. 2014a, Gaffney et al. 2015). Based on these examples, it is argued that we are now poised at a point where prediction of G × E × M interactions, and the contributions of G–M technology combinations to improved crop productivity provide the foundation for a feasible strategy to enhance and accelerate crop improvement and to close on-farm yield-gaps.

Beyond the specifics of predicting G × E × M interactions and selecting for G–M technology combinations, a general principle guiding development of future crop productivity prediction technologies is to develop the capability to identify crop improvement strategies that can avoid problems, before they arise, rather than solving those problems once they have occurred (Messina et al. 2020b). Here we consider the motivations for predicting G × E × M interactions from a perspective of identifying pathways to sustainable improvements in crop productivity, while avoiding those pathways that increase the risks of global food insecurity, given the environmental changes projected for climate change.

### Climate change and crop improvement

On a global scale, for recent periods, spatial patterns in associations between climate variability and crop yield productivity have been documented (Ray et al. 2015). The long-term trend of global increase in [CO_2_] is continuing (IPCC 2014; National Research Council 2020). Consequences of anthropogenic climate change for crop productivity have been the subject of recent investigations (Lobell and Burke 2010; Chapman et al. 2012; Lobell et al. 2015; Hammer et al. 2020; Chen et al. 2020). The projected increase in [CO_2_] is predicted to further contribute to a photosynthesis fertilization effect (Ainsworth and Long 2005; Ainsworth and Rogers 2007), reduced stomatal conductance and increased transpiration efficiency (Lobell et al. 2015; Leakey et al. 2019, Hammer et al. 2020). Experimental and modelling studies have demonstrated that the increases in [CO_2_] will have a positive photosynthesis fertilisation impact for C_3_ species (Ainsworth and Long 2005; Leakey et al. 2009). For C_4_ species, the impact will be through increased water use efficiency (Leakey et al. 2019). Associated increases in temperature will continue to have an impact on crop development patterns (Zheng et al. 2016). The consequences of accelerated development due to elevated temperatures will depend on the G × E × M opportunities within the context of the TPE (e.g. Hunt et al. 2019). The predicted increases in the frequency of high-temperature events, during critical yield determining stages of crop development, are likely to have significant negative consequences for yield productivity of all crops in future environments and will require coordinated adjustments in genotype and management technologies (Hammer et al. 2020).

Given the documented long-term trends in [CO_2_] and temperature, it is likely we are already dealing with their early effects on crop productivity in many of our crop improvement programs (Lobell and Field 2007; Chapman et al. 2012; Lobell et al. 2013, 2015; Hammer et al. 2014, 2020; Zheng et al. 2016; Snowdon et al. 2020). These effects have the potential to affect outcomes through their influences on evaluations of genotype adaptation and management strategies within multi-environment trials (METs). They have been investigated in terms of potential shifts in the environmental composition of the TPE (Lobell et al. 2015; Hammer et al. 2020). These studies demonstrated that near-term projected increases in temperature levels, their associated effects on crop water balance and the predicted increasing incidence of high-temperature events are of a sufficient magnitude to create shifts in the frequency of occurrence of major environmental conditions within a TPE (Chapman et al. 2012; Lobell et al. 2015; Hammer et al. 2020). Whenever the effects of climate change are of a sufficient magnitude to result in shifts in the environmental composition of the TPE, there will be the potential for associated shifts in the frequency of occurrence of different environment-types sampled within METs. The sampling strategy used by breeders and agronomists to design their METs will determine whether such climate change influences are assayed and can therefore be investigated and accounted for within the METs (Braun et al. 2010; Snowdon et al. 2020).

Given the challenges from the natural effects of climate variability and G × E interactions (Chapman et al. 2000, 2002), combined with anthropogenic climate changes (Chapman et al. 2012), prediction methodologies provide the potential for developing tools to identify new pathways for crop improvement that are responsive to the effects of climate change. Prediction methods provide tactical tools to choose among many options to achieve crop improvement and manage risk within the short to medium-term horizon (Hammer et al. 2014, 2020; Cooper et al. 2020). They can also provide strategic tools to define and navigate trajectories for long-term crop improvement (Messina et al. 2011) and sustainable intensification of crop systems (Cassman and Grassini 2020). To realise the potential of these opportunities, we require integrated modelling methods that can deal with the high dimensionality of the G × E × M problem space and can combine genomic prediction for breeding with agronomic prediction for management to improve crop productivity and reduce on-farm yield-gaps (Cooper et al. 2014b, 2020; Messina et al. 2018, 2020a, b; Hammer et al. 2020).

### Exploring the high dimensionality of G × E × M interaction space

With evidence of G × E × M interactions for traits relevant to crop productivity within a TPE, many questions require deeper consideration than they currently attract (Snowdon et al. 2020). An important question to consider is how to explore among the diverse genetic and management opportunities accessible within the high dimensionality of the complex G × E × M factorial, to identify those G–M technology combinations that can lead to sustainable improvements for crop productivity? This requires simultaneous consideration of contributions from each dimension, in order to avoid confounding the outcomes of the chosen crop improvement strategy with conditional effects that are associated with limited sampling of one or two of the other dimensions: the genetic dimension, in context with the reference population of genotypes (Messina et al. 2011; Ramstein et al. 2019), and the environmental dimension, in context with the TPE (Chapman et al. 2000; Lӧffler et al. 2005; Chenu et al. 2011; Kholová et al. 2013). The management dimension for its joint influences on both the environmental dimensionality and the contributions of trait genetic diversity to crop productivity (Chapman et al. 2003; Messina et al. 2011; Hammer et al. 2014; Snowdon et al. 2020). Thus, considering crop productivity from a G × E × M perspective, the actual dimensionality of the problem space is greater than expectations set by viewing the problem from only one of the contributing dimensions. To enable a foundation for prediction, we seek an understanding of important G × E × M interactions for crop productivity that can provide predictor variables for influential components of the genetic, environmental and management dimensions (Hammer et al. 2006; van Eeuwijk et al. 2019; Cooper et al. 2020; Hammer et al. 2020). We can then use these predictor variables to construct testable predictions to identify exploitable positive crop productivity outcomes from among the many possible G × E × M combinations (Cooper et al. 2002, 2020; Messina et al. 2009; Hammer et al. 2014, 2020; van Eeuwijk et al. 2019). To apply this foundation for G × E × M prediction, Messina et al. (2020a, b) proposed an iterative empirical-modelling approach based on a fusion of mechanistic biophysical models, statistical and machine learning approaches to explore the genetic, environment and management dimensions simultaneously. A novel component of their iterative empirical-modelling strategy was the use of suitably designed crop models, based on the ecophysiological principles of crop science, to supervise the simulation-based exploration of the G × E × M dimensionality for both genomic prediction (Technow et al. 2015; Messina et al. 2018; Diepenbrock et al. 2021) and agronomic prediction (Cooper et al. 2020; Hammer et al. 2020).

Building on the early demonstrations of successful applications of prediction methodology for maize G × E × M interactions in the US corn-belt, there are nascent opportunities emerging to consider broader applications for other crops and production systems. These developments are stimulating advances in the integrated approaches to crop modelling, phenotyping, machine learning and high-performance computing to harness “Big Data” from combinations of designed and on-farm empirical studies to enable prediction-based agriculture (Holzworth et al. 2014; Brown et al. 2014; Ramirez-Villegas et al. 2020; Casadebaig et al. 2020; Bogard et al. 2020; Sinclair et al. 2020; Ersoz et al. 2020; Washburn et al. 2020; Stöckle and Kemanian 2020; Cooper et al. 2021).

### Climate change and exploring the E dimension: non-stationary Target Population of Environments (TPE)

Beyond breeding for the average genetic merit of individuals for a TPE (e.g. Hallauer and Fo 1988; Comstock 1996), there is a long history of breeding for adaptation to specific environment-types encountered within a TPE (e.g. Blum 1988; Cooper and Hammer 1996; Bänziger and Cooper 2001; Gaffney et al. 2015). Comstock (1977) introduced the concept of a TPE for a breeding program. The TPE concept provides the environmental complement to the concept of a reference population of genotypes (RPG). Together, the RPG and the TPE provide a foundation for consideration of trait genetic architecture that takes into consideration G × E interactions within the target crop production system. At many stages of a breeding program, breeders conduct METs to evaluate samples of genotypes, from the RPG, in samples of environments, from the TPE. Breeders traditionally sample the environments of the TPE by distributing their trials across locations within a defined geography for a sequence of years. In the traditional view of G × E interactions (Comstock and Moll 1963), random G × E interactions, associated with the samples of environments realised in breeding METs, create an additional layer of uncertainty that complicates determining the genetic merit of individuals derived from the RPG (breeding value and genotypic value) (e.g. Hallauer and Fo 1988; Nyquist and Baker 1991; Comstock 1996, Fig. 1).

**Fig. 1:**
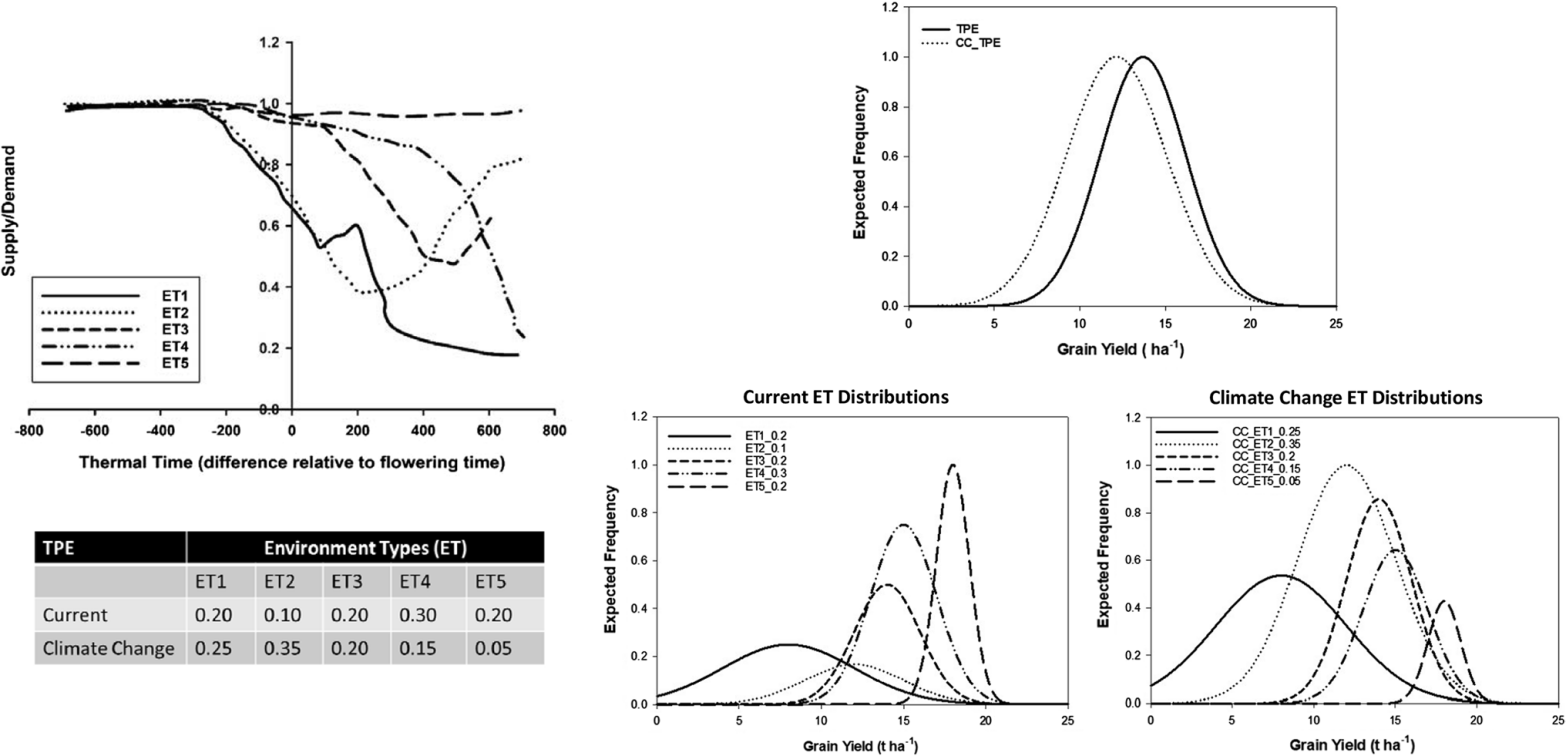
Schematic representation of the impact of projected influence of climate change (CC) on changes in the expected frequency of occurrence of five environment-types (ETs) and the associated changes in the distribution of observed crop grain yield productivity levels. Following the characterisation of the US corn-belt Target Population of Environments (TPE) and methodology reported by Cooper et al. (2014b), the depicted scenario represents a projection where there is an increase in frequency of occurrence of flowering and grain-filling water-deficit ETs (ET1 and ET2) and a decrease in frequency of occurrence of favourable ETs with low levels of water-deficit (ET4 and ET5). Characterisation of water-deficit is based on the water supply/demand ratio, relative to flowering time, estimated using a crop growth model

With advances in understanding of the genetic and environmental bases of G × E interactions, examples of breeding strategies targeting repeatable components of G × E interactions emerged (e.g. Blum 1988; Cooper and Hammer 1996; Bänziger and Cooper 2001; Braun et al. 2010; Windhausen et al. 2012; Gaffney et al. 2015). For many TPE scenarios, the typical sizes of METs and the traditional location-year sampling strategy can result in an inadequate representation of the mixture of environment-types that constitute the TPE (Chapman et al. 2000, 2002). Beyond the location-year sampling strategy, Comstock (1977, 1996) did not provide a formal treatment of how to apply the TPE concept for any components of the G × E interactions that could be associated with specific environmental targets, e.g. drought, temperature extremes, macro- and micro-nutrient availability, biotic stresses. However, breeding strategies designed to exploit components of genetic variation associated with G × E interactions require some level of consideration of how to characterise a TPE for the range of environmental conditions, particularly where this is not well represented by the location-year sampling strategy (Cooper et al. 1995; Chapman et al. 2002; Rebetzke et al. 2013). Inevitably, this requires decisions on the levels of granularity with which environments are distinguished. Approaches taken have ranged from coarse-grained to fine-grained characterisation of the environmental dimensions of the TPE. For example, targeting breeding for broad categories of environment-types, such as stress (abiotic or biotic) environments, represents a coarse-grained approach (e.g. Blum 1988; Snowdon et al. 2020). Subsequent developments of the original TPE concept considered the TPE as a mixture of repeatable environment-types (Atlin and Frey 1990; Cooper and DeLacy 1994; Cooper and Hammer 1996; Podlich and Cooper 1998; Chapman et al. 2000, 2002, 2003; Cooper and Podlich 2002; Cooper et al. 2005; Lӧffler et al. 2005; Chenu et al. 2011; Kholová et al. 2013). The definition of environment-types represents an example of a coarse-grained refinement of the TPE concept. As a first approximation of the structure of the TPE, definition of environment-types provides breeding targets for specific adaptations associated with identified repeatable sources of G × E interactions. Following this coarse-grained definition of the TPE, broad adaptation can be investigated in terms of performance across the environment-types (Podlich et al. 1999). Clearly, the breeder can decide on the appropriate level of granularity for characterising a TPE, from coarse-grained to fine-grained, depending on the level of understanding of the G × E interactions and the resources available to target breeding for specific environment-type targets (e.g. Messina et al. 2011, 2015; Windhausen et al. 2012; Gaffney et al. 2015; Kholová et al. 2013, 2014).

Further extensions of the formal TPE framework have also considered management as a subset of the environmental dimension (Messina et al. 2009; Hammer et al. 2014). Importantly, the definition of management strategies provides a range of suitable environmental descriptors to enable prediction that targets G × M interactions as a subset of the total G × E interactions. Applications towards this more fine-grained characterisation of the TPE have been demonstrated for a range of crops and geographies (e.g. Podlich et al. 1999; Chapman et al. 2000, 2012; Löffler et al. 2005; Chenu et al. 2011; Messina et al. 2011; Kholová et al. 2013; Cooper et al. 2014a; Hammer et al. 2014; Snowdon et al. 2020).

Building on the concept of a TPE for crop improvement, we consider potential applications for predicting G × E × M interactions while accounting for the effects of climate change (Chapman et al. 2012). Herein, the TPE for any crop improvement program defines the characteristics and frequencies of occurrence of the different environment-types, for the chosen level of granularity for characterisation, encountered within the spatial and temporal dimensions of the target agricultural systems (e.g. Fig. 1). The concept of a TPE applies broadly, from the diverse range of open systems that are common to field agriculture to the enclosed systems of high-value protected agriculture. In the presence of repeatable G × E interactions, breeding and trait performance prediction can be targeted at genetic differences for important repeatable environmental conditions within the context of the TPE (e.g. Chapman et al. 2003; Messina et al. 2011). Where important Genotype-by-Management (G–M) technology innovations provide opportunities to improve crop productivity for the environment-types of a TPE, the same principles applied to predict G × E interactions for breeding can be extended to predict G–M technology targets within the context of G × E × M interactions (Hammer et al. 2014, 2020; Cooper et al. 2020; Rotili et al. 2020). For this extension, selection would focus on the G–M technology combinations, directing coordinated improvement of genetics and management for the environment-types of the TPE.

Early applications of the TPE concept assumed a stationary target for a breeding program. However, today we recognise that the consequences of climate change contribute to a non-stationary TPE (Fig. 1, Braun et al. 2010, Chapman et al. 2012; Lobell et al. 2015; Hammer et al. 2020; Snowdon et al. 2020). When the concept of a TPE was first introduced, there was no consideration of the effects of climate change within the plant breeding literature. Today, motivated by research into the effects of climate change, we must accommodate both stationary and non-stationary targets within the formal treatment of the TPE for a breeding program. The non-stationary effects can be quantified in terms of changes in frequency of occurrence of the environment-types (Fig. 1). Further, we can extend the concept and the formal treatment to the broader context of crop improvement, taking into consideration G × E × M interactions for non-stationary environment-types changing in frequency of occurrence due to the effects of climate change (Chapman et al. 2012).

Successful crop improvement for climate resilience will require an understanding of the TPE and the environment-types for which new genotypes and agronomic strategies are to be developed. From the perspective of characterising a TPE, many of the changes associated with the natural and anthropogenic effects of climate change require consideration of the challenging non-stationary features of the TPE (e.g. Hunt et al. 2019; Choquette et al. 2019; Hammer et al. 2020; Chen et al. 2020; Snowdon et al. 2020). Further, without special attention to monitoring the key resources of the crop environment (e.g. temperature, [CO_2_], water, radiation, nutrition), increases in the concentration of pollutants (e.g. O_3_), or shifts in the incidence and severity of biotic stresses, many of the changes that can impact crop adaptation are likely to be imperceptible in the short term to the observer conducting METs for plant breeding or agronomic applications (e.g. Choquette et al. 2019). Consider the current rate of change in [CO_2_]. Chapman et al. (2012) emphasised the increase in [CO_2_] from the recent pre-industrial era (280 µmol mol^−1^) to the time of their publication (2012; 392 µmol mol^−1^), based on observations taken at the Mauna Loa Observatory located in Hawaii. In the intervening period, the monitored levels of [CO_2_] have increased further to 415 µmol mol^−1^ (January 2021; www.co2now.org). Hammer et al. (2020) considered how such increases in [CO_2_], combined with temperature increases and consequent changes in rainfall patterns, can influence the frequency of occurrence of different water–stress environment-types for sorghum in Australia. Without specifically asking these questions, the modelled changes in water–stress environment-types, and any associated G × E interactions, could appear as random background environmental variation to those responsible for conducting METs for the Australian sorghum breeding programs. These and other gradual background changes in frequencies of environment-types within a TPE, in combination with the regional and inter-annual variation in their occurrences, can act over multiple breeding program cycles to impact the trait adaptation requirements of crops for yield performance (Snowdon et al. 2020). Even if the direct effects of changes in [CO_2_] on breeding outcomes cannot be easily demonstrated (e.g. Ziska et al. 2004; Ainsworth and McGrath 2010), the indirect effects on season length (Hunt et al. 2019) and frequency of occurrence of water–stress events and temperature extremes (Hammer et al. 2020) can be demonstrated to impact the adaptation requirements of crops.

Within the context of the environmental variability expected for a TPE, the implementation of management strategies provides farmers some scope to reduce the impact of the environmental variation on crop productivity, at least relative to the expectations for the case where the environmental variability was uncontrolled by suitable agronomic management. The management interventions can be evaluated in terms of their influence on the environment-types of the TPE and their frequency of occurrence (Chapman et al. 2012; Lobell et al. 2015; Hammer et al. 2020). For example, in dryland and limited-irrigation production systems, reductions in plant density and adjustments in irrigation quantity and timing can both reduce the frequency of occurrence of water–stress environment-types within a TPE (e.g. Chapman et al. 2000; Chenu et al. 2011; Hammer et al. 2020).

Therefore, we argue that the prediction requirements for breeding climate resilient crops can be tackled by extending the definition of the characteristics of the TPE for a breeding program. In addition to defining the target environment-types that comprise the TPE, and their frequencies of occurrence in the current situation, there is a need to quantify the expected rates of change in their frequencies of occurrence that are a consequence of the effects of climate change (Fig. 1, Hammer et al. 2020). With an understanding of the rates of change in the environment-type targets, and an understanding of the trait requirements for adaptation and performance in the different environment-types, tactical and strategic breeding programs can be designed that prioritise breeding objectives with knowledge of the projected changes (Chapman et al. 2012; Hammer et al. 2020; Snowdon et al. 2020). Further, with appropriate attention to design, results from METs across multiple breeding program stages and cycles can be combined to conduct meta-analyses and develop robust training data sets to support genomic prediction (Cooper et al. 2014a, b). Through adequate sampling of the TPE and appropriate updating of the training data sets (e.g. Podlich et al. 2004), the design and conduct of METs provide a practical mechanism for addressing some aspects of the non-stationary effects of climate change on the environment-type composition of the TPE.

### Crop improvement and reducing yield-gaps

**Fig. 2:**
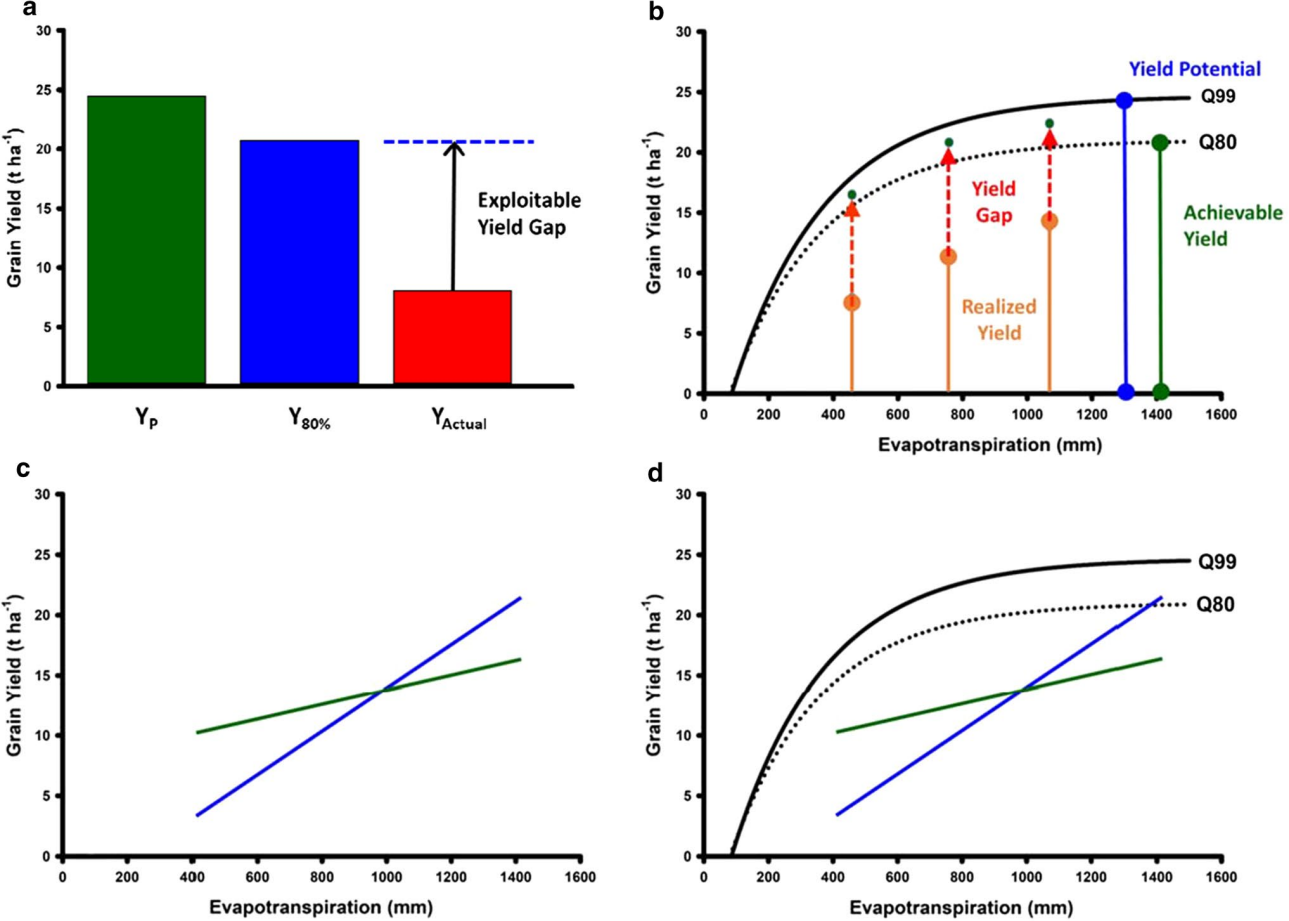
Schematic representation of an on-farm yield-gap from the perspective of an agronomist and a breeder: **a** Classical view of a yield-gap. *Y*_P_ defines the on-farm yield potential that can be expected when a suitable genotype is selected and all abiotic and biotic stresses are removed from the on-farm environment. *Y*_80%_ defines the yield level at 80% of the *Y*_p_. *Y*_Actual_ defines the actual on-farm yield that was achieved. The yield difference between the Y_80%_ and *Y*_Actual_ defines the exploitable yield-gap. **b** On-farm yield-gap depicted as a continuum of differences (as represented in sub-figure (**a**)), between the actual yield and target exploitable yield along the Yield-Evapotranspiration yield front. The target exploitable yield is defined in the example by the 99% Yield-Evapotranspiration front (Q99, Yield potential) and the 80% Yield-Evapotranspiration front (Q80, Achievable yield). **c** Classical plant breeding view of crossover Genotype-by-Environment interactions. **d** Plant breeding view of crossover Genotype-by-Environment interactions superimposed on the agronomist view of yield front

**Fig. 3:**
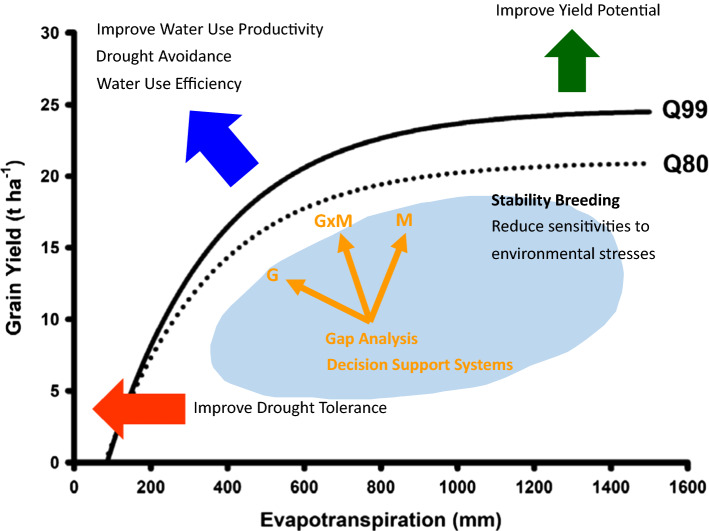
Schematic representation of plant breeding perspective on grain yield improvement considered in terms of improving Yield Potential, Effective Water Use, and Drought Tolerance in relation to enhancing the yield front. Breeding for yield stability can be considered in terms of breeding for trait combinations that improve genotype yield performance and reduce the yield-gap along the continuum of the yield front. Gap analysis and reduction of yield-gaps can then be investigated in terms of selection of genotypes, management strategies and genotype–management technology combinations to reduce the gap between actual on-farm yields and achievable yield levels along the yield front continuum

Improvements in on-farm crop productivity can be evaluated in terms of the genetic improvement of crop yield potential, given the resource inputs of the target agricultural system, and genetic improvement of yield stability to reduce the gap between the realised on-farm productivity and the expected yield potential (Fig. 2a; van Ittersum et al. 2013; Fischer et al. 2014). For any given TPE, the concept of defining yield potential, achievable yield and yield-gaps can be quantified in terms of defining the yield front, given the definition of the limiting environmental resources (e.g. French and Schultz 1984; Sadras et al. 2015; van Bussel et al. 2015; van Oort et al. 2017); e.g. the water-limited yield front for maize in the US corn-belt (Fig. 2b; Cooper et al. 2020). Plant breeders have focused on genetic improvement of yield potential and yield stability for a TPE through the design of breeding programs (Hallauer and Fo 1988; Comstock 1996). There have been significant investments in technology development to accelerate rate of genetic gain (Voss-Fels et al. 2019) for important traits contributing to yield potential and yield stability for the combinations of abiotic and biotic stresses that are encountered within the TPE. Plant breeders are interested in the potential for G × E interactions to change the rank order of genotypes between the important environment-types of a TPE (e.g. Fig. 2c). Through breeding for yield stability, breeders seek to close the yield-gap between the current on-farm yield level and the potential yield level by selecting new genotypes with new combinations of the alleles for the genes influencing the levels of trait expression resulting in yield levels closer to the yield potential front. Agronomists have focused on the development of crop management strategies that improve the effective use of environmental resources (e.g. radiation, temperature, water, nutrition), inputs (e.g. fertiliser, herbicides, pesticides and water through irrigation), and farmer management decisions (e.g. planting time, plant density, plant spacing configurations and tillage practices). They seek management strategies that increase the likelihood of achieving crop yield targets (e.g. breakeven yield, achievable yield and yield potential) for the range of environment-types within the TPE for the currently available set of genotypes. Typically, the breeder and agronomist views are considered separately. However, they could be combined into one view (e.g. Fig. 2d). With such a combined view, it is possible to explore integrated crop improvement strategies that seek to close the gap between the current yield levels and the achievable and potential yield levels. Such an integrated approach could be enabled through selection for combinations of genetic and agronomic improvement, ultimately taking advantage of favourable G–M combinations that deliver on-farm yield productivity closer to the potential for the range of environments of the TPE (Fig. 3).

### From describing G × E × M to predicting (G–M) × E interactions

To date, the majority of investigations of G × E × M interactions for crop improvement have involved describing the major features within accessible data sets, and testing whether significant G × M interactions are present. When significant G × M and G × E × M interactions are found to be present, further studies have provided new lists of the importance of traits and trait target recommendations for plant breeders. In some cases, multiple trait combinations are used to define ideotypes as potential new breeding targets. Occasionally, the new breeding targets are defined in combination with new agronomic practices, for example selection for genotypes that tolerate the specific abiotic and biotic stress consequences associated with growing crops at high plant density in combination with recommendations for the higher plant density targets and also alternative row configuration and fertiliser recommendations (e.g. Hammer et al. 2014, 2020; Gaffney et al. 2015). Research undertaken to define new breeding and agronomic targets in response to G × E × M interactions indicates that the current research methods, based on breeding followed by management optimisation, are likely to be inefficient or incapable of creating the proposed new G–M combinations. This may be the case for the consequences of climate change.

Many of the predicted changes in the structure of the TPE, as a consequence of climate change scenarios, are anticipated to impact the relative importance and contributions of traits to crop yield productivity (e.g. see multiple chapters in Yadav et al. 2011; Chapman et al. 2012; Hammer et al. 2020; Snowdon et al. 2020). These situations pre-empt changes in the trait targets required to sustain genetic gain for crop productivity. Similarly, the relative merits of alternative management strategies may also be anticipated to change with the structure of the TPE as a consequence of climate change. Thus, we can anticipate that there is the potential for changes in the importance and the features of the resulting G × E × M interactions for crop productivity in a TPE that is shifting under the influence of the effects of climate change. If such a situation unfolds, then describing G × E × M interactions will be inadequate for developing the required climate resilient crops for the future and for the design of suitable crop improvement strategies to deliver the required climate resilient crops. An alternative strategy is to focus on research methodologies to identify G–M technology combinations (Hammer et al. 2014; Messina et al. 2020b). In this case, the prediction targets and the selection units of the crop improvement strategy would be the G–M combinations that result in improved on-farm crop productivity. This would represent a shift towards prediction of productive G–M combinations for the current and predicted future TPE (Hammer et al. 2020). Thus, instead of describing G × E × M interactions the focus would shift to predicting G × M interactions for different G–M technology combinations and quantifying their performance characteristics for the environment-types of the TPE, herein defined as predicting (G–M) × E interactions, following Messina et al. (2020b).

Hammer et al. (2014) proposed focusing prediction on identifying G–M technology combinations to enhance crop improvement and risk management and demonstrated applications for sorghum crop improvement in Australia. Similarly, for maize in the US corn-belt, Cooper et al. (2014b) advocated G × E × M modelling throughout the breeding program cycle to augment the empirical testing footprint of METs. Recent examples have demonstrated applications of these proposals for sorghum in Australia (Hammer et al. 2020) and maize in the US corn-belt (Cooper et al. 2020).

### Predicting (G–M) × E interactions: an example

A maize example from the US corn-belt, where G × E × M interactions for yield under drought are important, is used to emphasise important considerations and demonstrate opportunities and potential approaches to prediction for crop improvement in the presence of G × E × M interactions. We consider a subset of the simulated grain yield (GY) and crop evapotranspiration (ET) results reported by Cooper et al. (2020). Within their larger study, they contrasted G × E × M interactions for GY and ET between two major regions of the US corn-belt: the Western region, represented by a location in Kansas, and the Central region, represented by a location in Iowa. As background to the example, these two regions of the US corn-belt are expected to have different levels of G × E × M interactions for GY associated with differences in ET at the environmental, management, and genotypic levels. In the Western region, rainfall is low and vapour pressure deficit is high and dryland maize GY is strongly water limited. Irrigation and plant density are important management strategies used by farmers to avoid or minimise yield reductions due to water limitations, i.e. to reduce the yield-gap (Figs. 2 and 3). Consequently, in the Western region, differing patterns of crop water use and ET, associated with differences in genotype and management, are expected to have a strong influence on G × E × M interactions for GY across locations and years (Gaffney et al. 2015). In contrast in the Central region, rainfall is higher and vapour deficits are lower and water limitations are less frequent. Consequently, in the Central region, differences in patterns of water use and ET, associated with genotype and management, are expected to have less influence on G × E × M interactions for GY across locations and years (Gaffney et al. 2015). The simulated ET and GY data subset considered here was centred on the same Kansas and Iowa locations considered by Cooper et al. (2020). The number of locations was expanded by selecting eight additional surrounding locations within each region. Thus, nine locations from both Kansas and Iowa were identified. For each location, simulated GY and ET data were generated for twenty sequential years (1996–2015). For the purposes of the analyses reported here, the focus was on contrasting G × E × M interactions between Kansas and Iowa, representing a contrast between the Western and Central regions of the US corn-belt. Following Cooper et al. (2020), the twelve management strategies considered were based on the same factorial combination of three plant densities (6, 8 and 10 plants m^−2^) and four irrigation strategies: no supplementary irrigation (NI), fully irrigated (FI), one 20 mm irrigation at V12 (V12), weekly irrigation to replace cumulative ET loss in the prior week (WI). The GY and ET data were simulated for all Location, Management and Year combinations for the same 488 genotypes using the same crop model considered by Cooper et al. (2020). In summary, the simulated genotypes were based on all possible combinations of three different levels of expression of five traits, which were previously identified to influence GY of elite maize hybrids across US corn-belt environments (Messina et al. 2018): leaf number and leaf size, which together determined potential canopy size, canopy radiation use efficiency, canopy level limited transpiration, and reproductive resiliency, which determined silk exertion rate, anthesis to silking interval and impacted kernel set and GY. Two levels of maturity were also considered for each genotype. One check hybrid with two maturity levels was also included.

Analyses of variance of the complete simulated data set emphasised the importance of G × E × M interactions for GY and the important role of water availability. The contributions of the different water availability environments and the associated crop water use outcomes of the genotype and management dimensions for the range of environments were represented by the simulated ET trait. Within the full data set, there was a strong contrast in the G × E × M interactions for the GY results between Kansas and Iowa. Therefore, for demonstration purposes here, the simulated GY and ET results for the G × E × M combinations were analysed separately for Iowa and Kansas to estimate variance components and compute best linear unbiased predictors (BLUPs) based on the model:

1 $$T_{ijkl} = \mu + y_{j} + m_{k} + \left( {ym} \right)_{jk} + r_{l} + g_{i} + \left( {gy} \right)_{ij} + \left( {gm} \right)_{ik} + \left( {gmy} \right)_{ijk} + \varepsilon_{ijkl} ,$$

where *T*_*ijkl*_ is the simulated trait value (GY or ET) for genotype *i*, in year *j*, management *k* and location *l*, *μ* is the overall mean, *y*_*j*_ is the effect of year *j*, *m*_*k*_ is the effect of management *k*, *(ym)*_*jk*_ is the interaction effect for year *j* and management *k*, *r*_*l*_ is the effect of location *l*, where the locations were considered as nine replicates within each of Kansas and Iowa, *g*_*i*_ is the effect of genotype *i*, *(gy)*_*ij*_ is the interaction effect of genotype *i* and year *j*, *(gm)*_*ik*_ is the interaction effect of genotype *i* and management *k*, *(gmy)*_*ijk*_ is the interaction effect of genotype *i*, management *k* and year *j*, and *ε*_*ijkl*_ is the residual effect. All terms, except *μ*, were assumed to be random, normally distributed variables. For both Iowa and Kansas, GY and ET BLUPs were computed for the 12 management levels (M_BLUPs), for the 488 genotypes (G_BLUPs), and for the 5856 G–M technology combinations (GM_BLUPs).

**Fig. 4:**
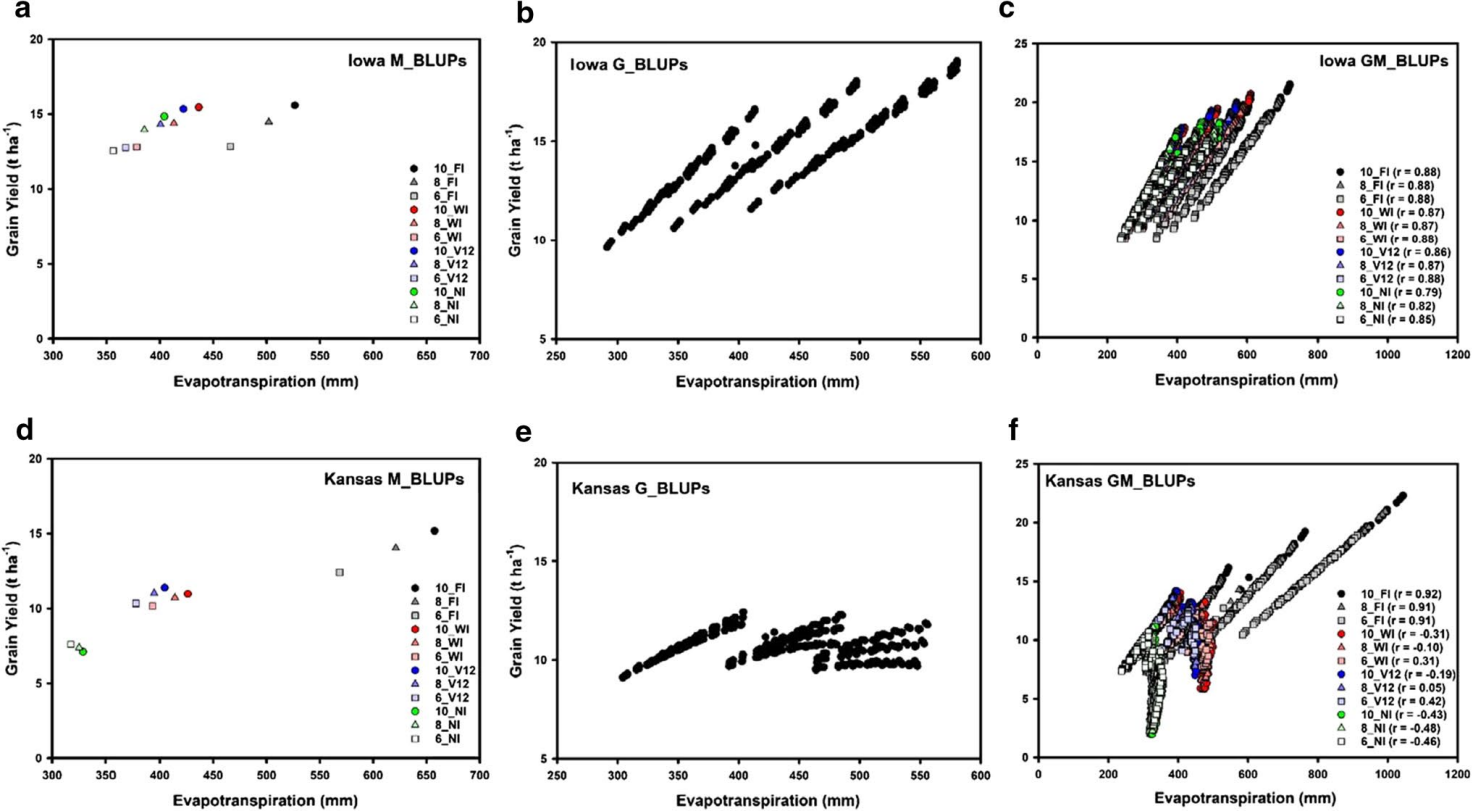
Comparison of grain yield and evapotranspiration BLUPs for maize Genotype-by-Environment-by-Management (G × E × M) interaction case study: **a** Iowa Management BLUPs (M_BLUPs), **b** Iowa Genotype BLUPs (G_BLUPs), **c** Iowa Genotype–Management Technology BLUPs (GM_BLUPs), **d** Kansas Management BLUPs (M_BLUPs), **e** Kansas Genotype BLUPs (G_BLUPs), **f** Kansas Genotype–Management Technology BLUPs (GM_BLUPs)

**Fig. 5:**
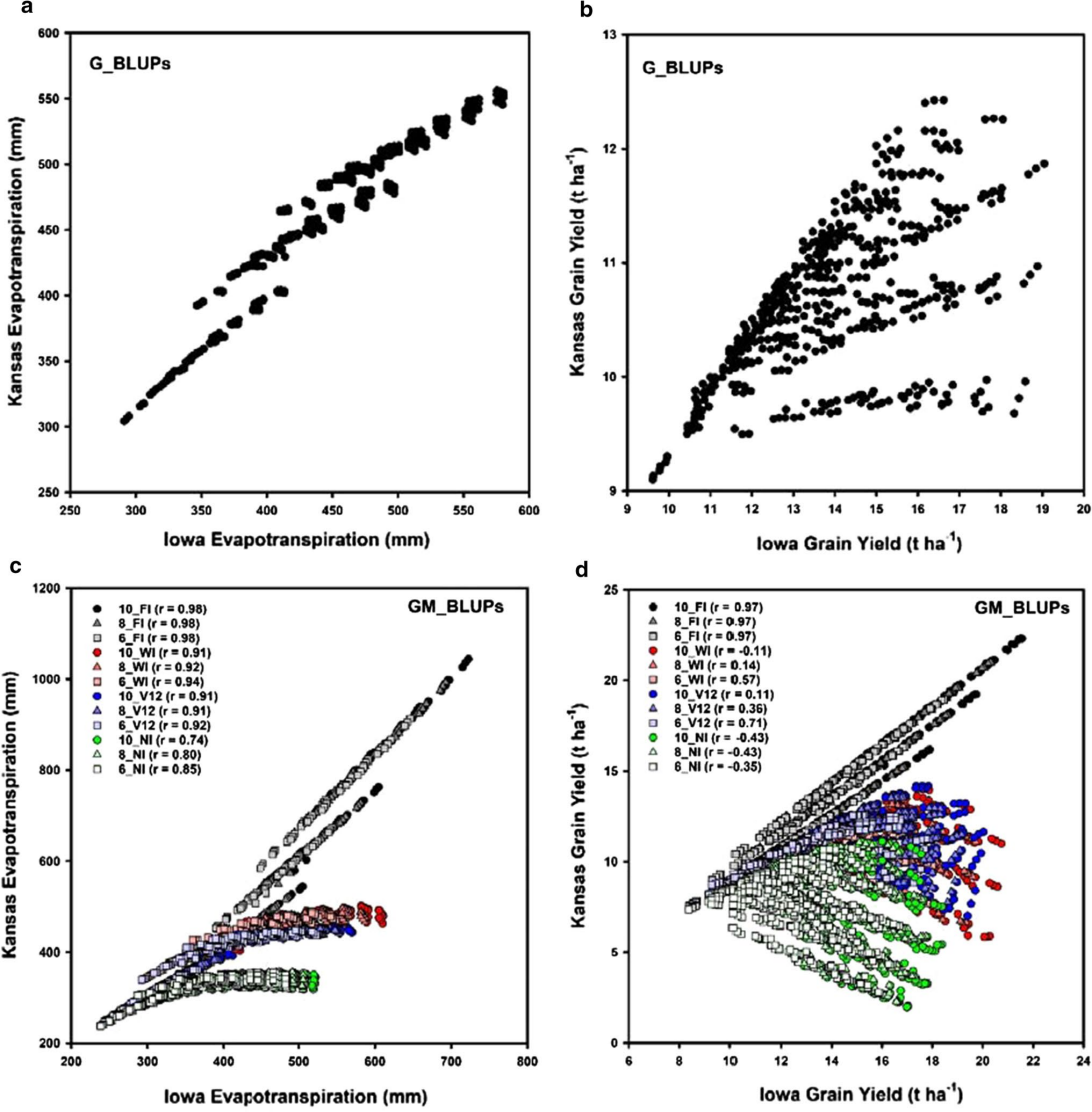
Comparison grain yield and evapotranspiration BLUPs between Kansas and Iowa for maize Genotype-by-Environment-by-Management (G × E × M) interaction case study: **a** Evapotranspiration Genotype BLUPs (G_BLUPs) compared between Kansas and Iowa, **b** Grain Yield Genotype BLUPs (G_BLUPs) compared between Kansas and Iowa, **c** Evapotranspiration Genotype–Management Technology BLUPs (GM_BLUPs) compared between Kansas and Iowa, **d** Grain Yield Genotype–Management Technology BLUPs (GM_BLUPs) compared between Kansas and Iowa

For both Iowa and Kansas, the location source of variance was small for both GY and ET (Table 1). The year source of variance was larger for Kansas than Iowa for both GY and ET, although these were relatively small compared to other sources of variance (Table 1). For Iowa, the genotypic component of variance was the largest component for both GY and ET (Table 1). In contrast, for Kansas, the management component of variance was larger than the genotypic and Genotype-by-Management components of variance for both GY and ET (Table 1). There were relationships between GY and ET for both Kansas and Iowa (Fig. 4). The GY-ET relationships differed, depending on both location (Iowa (Fig. 4a–c) or Kansas (Fig. 4d–f)) and prediction level: M_BLUP (Fig. 4a, d), G_BLUP (Fig. 4b, e), or GM_BLUP (Fig. 4c, f). For Kansas, the GY Genotype-by-Management interaction variance component was larger than the genotypic variance component, while smaller for ET (Table 1). In contrast, for Iowa the genotypic component was larger than the Genotype-by-Management interaction component for both GY and ET (Table 1). Notably, the magnitude of the ET genotypic variance components was similar for both Iowa and Kansas, while the magnitudes contrasted for GY. Further, the G_BLUPs for ET were similar between Iowa and Kansas (Fig. 5a). The consistency in total water use by the genotypes, indicated by the consistent ET (Fig. 5a), in combination with the different environmental conditions between Iowa and Kansas, resulted in contrasting G_BLUPs for GY between Iowa and Kansas (Fig. 5b). The differences in environmental conditions between Kansas and Iowa also resulted in contrasting GM_BLUPs for ET (Fig. 5c) and GY (Fig. 5d). These resulting contrasts in magnitude and structure of the Genotype-by-Management interactions for ET and GY between Kansas and Iowa provide the foundation for a suitable case study to consider issues involved in prediction of G × E × M interactions.

**Table 1:** Estimated variance components and their standard errors for grain yield and evapotranspiration based on a simulated maize data set

Source	Iowa		Kansas	
	Grain yield	Evapotranspiration	Grain yield	Evapotranspiration
	t ha^−1^	mm	t ha^−1^	mm
Location	0.14 ± 0.070	170.1 ± 85.0	0.05 ± 0.025	34.0 ± 17.0
Year (Y)	0.77 ± 0.252	297.6 ± 101.0	2.72 ± 0.912	1709.6 ± 590.8
Management (M)	1.27 ± 0.543	2813.7 ± 1203.3	6.31 ± 2.718	13,468.4 ± 5774.3
Y × M	0.14 ± 0.013	160.5 ± 15.7	1.13 ± 0.111	1325.0 ± 129.7
Genotype (G)	4.18 ± 0.269	5090.6 ± 327.0	0.68 ± 0.061	4807.0 ± 327.3
G × Y	0.13 ± 0.002	78.9 ± 1.3	0.43 ± 0.007	207.6 ± 3.6
G × M	0.09 ± 0.002	86.9 ± 1.8	2.95 ± 0.058	3454.5 ± 67.2
G × M × Y	0.11 ± 0.001	83.5 ± 0.6	0.44 ± 0.003	355.1 ± 2.0
Residual	0.42 ± 0.001	400.4 ± 0.6	2.04 ± 0.003	773.9 ± 1.1

The simulation study was designed to represent a sample of the Genotype, Environment and Management dimensions for Iowa, representing the Central region of the US corn-belt, and Kansas, representing the Western region of the US corn-belt

Selection for high GY, based on G_BLUPs, identified different genotypes depending on whether the selection was conducted within Iowa or Kansas (Fig. 5b). Selection for high GY, based on G_BLUPs, in Iowa identified genotypes with a wide range of GY G_BLUP values in Kansas (Fig. 5b). Therefore, based on the simulated influences of the five traits on the patterns of water use and their consequences for GY, multiple contrasting trait combinations resulted in high ET and high GY for Iowa. However, not all of these trait combinations that contributed to high GY for Iowa resulted in high GY for Kansas. If selection was conducted at the GM_BLUP level, there were contrasting G–M technology combinations that contributed to high GY for Iowa and Kansas (Fig. 5d). However, when selection was conducted for GY at the GM_BLUP level the same G–M technology combinations were identified to result in the highest GY levels for both Iowa and Kansas (Fig. 5d). Therefore, for both Iowa and Kansas, if water limitations were avoided, applying the FI management, the same G–M technologies were identified. However, if the FI management option was not available then different G–M technology combinations resulted in high GY for the Iowa and Kansas Locations. Further, the simulated GY results indicate that depending on the management strategy selected, the correlation for the GY GM_BLUPs between Iowa and Kansas could range from positive (e.g. 10_FI, Fig. 5d) to negative (e.g. 10_NI, Fig. 5d). Thus, strong crossover G × E interactions can occur for GY between Iowa and Kansas. However, with an understanding of the biophysical bases of the crossover G × E interactions for GY (e.g. Figs. 4 and 5) it is possible to deconvolute the contributing environmental and management conditions, here in terms of their impact on ET, and identify the contributing genotypic, management and environmental discriminating variables. With such an understanding of the discriminating variables, suitable genotypic and management predictor variables can be defined and an informed approach to design and prediction of G–M technologies for different target environments can be developed, using the crop model and GM_BLUPs in this example. The example discussed here used simulation results to demonstrate the application of the principles. The coordinated application of simulation and empirical studies can be undertaken in an iterative empirical-modelling process (Cooper et al. 2002; Messina et al. 2009, 2020a, b). Based on the understanding of G × E × M interactions for crop performance indicators, as quantified in the crop model, a comprehensive evaluation of G–M technologies can be first conducted using simulation. Promising G–M technologies identified by simulation can then be prioritised for empirical testing. Such iterative empirical-modelling applications were used to guide development of maize hybrids with improved yield stability for a wide range of drought environments and responsiveness to inputs for favourable environments, based on these same principles, for the US corn-belt (Campos et al. 2004; Barker et al. 2005; Messina et al. 2009, 2011, 2018, 2020a; Cooper et al. 2014a, 2016, 2020; Gaffney et al. 2015; McFadden et al. 2019).

### Exploiting (G–M) × E interactions: creating new pathways to accelerate crop improvement

Improving our understanding of the importance and influence of G × E × M interactions for on-farm yield productivity can open new opportunities for crop improvement to both improve yield potential for the range of environments of a TPE and to improve yield stability contributing to reduced yield-gaps between improvements in yield potential and on-farm yield (Fig. 3). Here we can highlight such opportunities for two broad crop improvement strategies.

First, within the current dominant crop improvement paradigm of first breeding to develop new cultivars followed by management optimisation. To date, most considerations of G × E × M interactions for crop improvement have operated from assumptions based on this serial, breeding followed by agronomy, perspective (Beres et al. 2020; Snowdon et al. 2020). For example, with advanced understanding of G × E × M interactions, breeders can adjust the design of the METs for one or more stages of the breeding program to enhance testing under different crop management regimes at any stage of the breeding program to create, identify and select new genotypes that demonstrate broad or specific adaptation to Environment–Management combinations that are important within the context of the TPE (Braun et al. 2010; Chapman et al. 2012; Snowdon et al. 2020). This strategy can be expected to broaden the range of environment-type contexts that genotypes are tested within, prior to their advancement and commercialisation for use by farmers. Further, using the same G × E × M understanding, agronomists could consider advanced testing of pre-commercial and new-commercial cultivars for optimisation of management to minimise the yield-gaps between yield potential and on-farm performance of the new cultivars when these are available to farmers (Rotili et al. 2020). This would also provide advanced access to the appropriate and potential future Environment–Management combinations expected to be used by farmers. Such advanced knowledge provides feedback to breeders to guide design and refinement of the MET stages of breeding programs (Messina et al. 2020a). Breeders could in turn explore potential sources of novel genetic variation for traits contributing to improved performance in projected future Environment-Management targets (Hammer et al. 2020; Snowdon et al. 2020).

Second, knowledge of G × E × M interactions can also open new opportunities for novel crop improvement strategies that target improvement of G–M technology combinations as integrated selection targets. Targeting G–M technology combinations as the units of selection creates opportunities to exploit positive G × M interactions for the environment-types of the TPE. To date less attention has been given to considering G × E × M interactions and their implications for crop improvement from this perspective. Here we have proposed that advances in simulation modelling (Messina et al. 2009, 2020a, b; Hammer et al. 2019, 2020; Peng et al. 2020) and prediction methodologies for both breeding (Heffner et al. 2009; Cooper et al. 2014b; Technow et al. 2015; Voss-Fels et al. 2019; Diepenbrock et al. 2021) and agronomy (Hammer et al. 2014, 2020) open new opportunities for undertaking such investigations of alternative crop improvement strategies. Suitable crop modelling platforms for a wide range of agricultural systems are available (Hammer et al. 2010; Holzworth et al. 2014; Brown et al. 2014). Key crop production system hallmarks that could be examined to justify considering investigations to refocus crop improvement strategies to exploit (G–M) × E interactions and their understanding include:

- Presence of strong G × E × M interactions for GY, with a significant G × M interaction component and identifiable G–M technology combinations that can be targeted as a unit of selection in a crop improvement strategy.
- Appropriate levels of environmental characterisation can be achieved to enable reliable prediction of the preferred G–M technology combinations for the expected on-farm environments.
- The different positive levels of G–M technology combinations can be targeted to appropriate on-farm environments within the range of important environment-types of the TPE.
- Farmers have access to the resources required to adopt the recommended G–M technology combinations for their on-farm environmental conditions.

Here we have argued that if we can understand and predict the performance properties of G–M technology combinations within the context of G × E × M interactions, we can potentially open up new crop improvement strategies to accelerate improvements in on-farm crop productivity. Given the historical contributions of both genetics and agronomy to improvements in on-farm crop productivity, there is strong justification for investigating the potential of applying prediction methods to focus some of our future crop improvement investments into the design and evaluation of strategies to explore and exploit G–M technology combinations. The predicted effects of climate change on the future TPE for crop improvement further motivate the need for such investigations (Chapman et al. 2012).

#### Targeting (G–M) × E interactions: implications for crop improvement

As with the presence of G × E interactions, the presence of G × E × M interactions has implications for the effective design of crop improvement strategies. However, there are some important differences. The presence of G × E × M interactions requires specific consideration of the coordinated improvement of both crop genetics and crop management to improve on-farm crop productivity. An important question we have emphasised here is whether the same workable G–M technology solutions can be discovered through continuation of, or the refinement of, the current dominant serial crop improvement approach, breeding followed by agronomic optimisation, and alternative crop improvement strategies that are specifically designed to select directly for G–M technologies simultaneously at all stages. This question can be investigated in terms of the opportunities to improve both the yield productivity front, within the context of a TPE, and reduce the yield-gap between the on-farm crop productivity and the productivity potential defined by the yield productivity front (Fig. 3). Given the many potential influences of climate change on the sustainability of the current levels of crop productivity and the projected future needs for global food security, seeking answers to this question is becoming increasingly urgent. Given the absence of comprehensive information on the magnitude and form of G × E × M interactions for yield and quality for many crops, cropping systems and TPEs, and the lack of any extensive empirical studies comparing such alternative crop improvement strategies, simulation methodology provides a viable approach for investigation of the appropriate design of crop improvement strategies in the presence of G × E × M interactions (Messina et al. 2020b; Hammer et al. 2020; Peng et al. 2020). Specific areas requiring particular attention in research programs can be identified:

- As with all evolving biophysical systems, for agricultural systems, improved performance outcomes are a consequence of the interplay between the genetic variation for traits, the characteristics of the environments within which the traits are expressed, and importantly the relative rates of change in the variables determining the environmental context to which the performance contributions of traits are determined. This interplay of the genetic and environmental dimensions can be understood as a performance landscape, or response surface (Gavrilets 2004; Cooper et al. 2005; Messina et al. 2011; Walsh and Lynch 2018). The shape of the performance landscape can change in response to changes in the environmental contexts that dominate the TPE and the influences of the traits that have been exploited over the history of the agricultural system (Messina et al. 2009, 2011; Chapman et al. 2012). Quantifying the rates of change of the variables involved in the environmental dimension of the G × E × M systems, as a consequence of climate change, requires increased attention as an integral component of characterising the target environments and the TPE of crop improvement programs.
- Crop improvement programs should be understood as search strategies that can be designed to explore the performance landscapes of the G × E × M systems of agriculture (Cooper and Podlich 2002; Messina et al. 2011). Their design has an influence of the properties of the landscape that can be explored and exploited and thus the workable G–M technology solutions that can be discovered (Cooper et al. 2005).
- Greater consideration should be given to undertaking integrated breeding and agronomy METs that are designed to test for the presence of G × E × M interactions, to quantify their magnitude and to characterise their structure. Such integrated METs would provide access to the required data to enable the iterative empirical-modelling approaches discussed here. The implications of climate change gives increased urgency to this requirement.
- Given the potentially high dimensionality of the G × E × M factorial it is likely that many workable G–M technology solutions will require evaluation (e.g. Fig. 5). Therefore, the current research emphasis on defining ideotypes as targets for breeding programs should be reformulated to define cohorts of potential workable G–M technology solutions for testing in the current dominant environment-type targets and also for the predicted future environment-type targets under the projected climate change scenarios. The iterative empirical-modelling framework discussed here would enable such a broadening of the design and optimisation of crop improvement strategies in the presence of important G × E × M interactions.
- Greater consideration should be given to the design of crop improvement strategies that focus on selecting for G–M technology targets. Large commercial breeding organisations have the resources to undertake such comparisons of alternative crop improvement strategies and to resource their practical implementation (e.g. Messina et al. 2020a). With sufficient experience, and empirical results obtained from crop improvement programs designed to select for G–M technologies, comparisons can be made between the rates of improvement in on-farm crop productivity that can be achieved by directly selecting for G–M technologies relative to the current rates of improvement that are being achieved (e.g. Fisher et al. 2014; Smith et al. 2014; Snowdon et al. 2020) from the dominant serial, breeding followed by agronomic optimisation, crop improvement methodology.
- Powerful prediction-based crop improvement strategies are emerging. Two advantages were emphasised here. Firstly, they provide a viable approach to tackle the high dimensionality of the G × E × M factorial (Messina et al. 2020b). Attention should be given to approaches that combine empirical studies with the strengths of predictive breeding and predictive agronomy for both tactical and strategic applications. Secondly, they provide practical and affordable pathways to support an upscaling of crop improvement programs when the cost of increasing the size of the empirical crop improvement program is beyond the reach of the program.
- For the complex traits that are the targets of crop improvement programs we can anticipate that prediction methods based on a fusion of mechanistic crop models and machine learning, which both include trait genetics, will be required (Messina et al. 2020b; Ersoz et al. 2020; Washburn et al. 2020; Diepenbrock et al. 2021).
- Optimisation of prediction-based crop improvement strategies will require a continuous iterative empirical-modelling approach. Within the iterative cycles the empirical breeding and agronomy programs will take on a new role. They will be embedded within the prediction-based programs and provide the critical data for design of appropriate sequences of training data sets to parameterise, evaluate and improve the prediction models.
- Telling the breeder or the agronomist to change their current approaches to crop improvement will not, by itself, catalyse the required change, even when the methodology has been convincingly demonstrated in a robust research setting. There is much history in the crop improvement literature that shows recommendations by themselves will not bring about the needed change. Real impact will require a more sustained transdisciplinary effort (Hammer et al. 2019). This will take time and most likely generations of crop improvement researchers, with new skills introduced over iterative cycles. This long-term perspective is consistent with the long history of design, testing, learning and implementation that underpins successful crop improvement outcomes. For example, the maize hybrid breeding methods that we are familiar with today were built on such a long history (Duvick 2005a, b; Technow et al. 2020) and continue to be refined (Cooper et al. 2014b; Messina et al. 2020a).

Whenever research demonstrates opportunities for new pathways towards enhanced climate resilient crop productivity, the translation of an innovation into a truly game-changing approach, which can accelerate crop improvement towards more sustainable crop productivity outcomes in the face of climate change, will require a sustained effort to integrate the new methodology into the core crop improvement processes over multiple iterative cycles (Vermeulen et al. 2018). This long-term perspective should underpin any research efforts that seek to deliver crop productivity outcomes in the form of climate resilient crops for the future.
